# The Application of a Mathematical Model to Evaluate the Effectiveness of Control Strategies Against *Ciona intestinalis* in Mussel Production

**DOI:** 10.3389/fvets.2019.00271

**Published:** 2019-08-20

**Authors:** Thitiwan Patanasatienkul, Javier Sanchez, Jeff Davidson, Crawford W. Revie

**Affiliations:** ^1^Department of Health Management, Atlantic Veterinary College, University of Prince Edward Island, Charlottetown, PE, Canada; ^2^Department of Computer and Information Sciences, University of Strathclyde, Glasgow, United Kingdom

**Keywords:** mathematical model, *Ciona intestinalis*, tunicates, treatment effectiveness, optimization, mussel

## Abstract

The Prince Edward Island (PEI) mussel industry has faced challenges associated with invasive tunicate species over the past two decades. Field experiments to find suitable mitigation strategies require considerable time and are resource intensive. This study demonstrates the application of a mathematical model to assess several control strategies against *Ciona intestinalis* populations under different temperature conditions in a mussel production area in PEI. A temperature dependent compartmental model was used to model the total abundance of *C. intestinalis*. Each mitigation strategy was defined in terms of a combination of timing and frequency of treatments. Various strategies were explored to obtain the combination that maximized the difference in predicted abundances between the control (untreated) and the different mitigation strategies. Treatment frequency was allowed to vary between one and four times over a given production year. The model was assessed under baseline conditions, which mimicked water temperatures from Georgetown Harbor, PEI, in 2008; as well as under scenarios that reflected prolonged summer or warm spring temperatures. Furthermore, the sensitivity of the model to variations in presumed treatment efficacy was evaluated. The use of all four available treatments, starting around the first week of July and correctly timed thereafter, provided the most effective strategy, assuming the baseline temperature scenario. However, the effectiveness of this mitigation strategy depended on temperature conditions. The mathematical model developed in this study allows decision makers to explore different strategies to control the abundance of *C. intestinalis* in mussel production areas under different environmental conditions. In addition, the modeling framework developed could be adapted to simulate comparable ectoparasitic infestation in aquatic environments.

## Introduction

The blue mussel (*Mytilus edulis* Linnaeus, 1758) accounts for around 40% of the total value of Canadian shellfish production, at around $38 million (1). In 2016, PEI was the main producer, responsible for 80% of all mussel production in Canada, corresponding to 19,732 tons (1). Over the past two decades, the PEI mussel industry has been challenged with the infestation of aquatic invasive species, particularly tunicates, which foul mussel socks and culture gear, causing significant economic losses to the industry due to the added production costs of biofouling control during grow-out and labor at processing plants (2–4).

Several invasive tunicate species have been identified in PEI waters, including the vase tunicate (*Ciona intestinalis* Linnaeus, 1767), which has most impacted PEI mussel production (3, 5). *C. intestinalis* is a fast growing, solitary tunicate, with a short-lived planktonic stage, which becomes a sessile filter feeder after settling and metamorphosis (3). This biofouling species grows very quickly when the sea water temperature is warm, while the growth rate declines with decreasing temperatures (3, 6). Because of the rapid growth of *C. intestinalis* populations, a mussel sock can become infested with a heavy tunicate biomass over the course of a few weeks, compromising the mussels' attachment to the socking material, resulting in mussel loss due to fall-off when socks are lifted (7).

Three mitigation techniques are commonly used to remove tunicates from mussel socks and aquaculture gear: chemical, natural, and mechanical methods. Chemical methods include calcium hydroxide (hydrated lime) for *Styela clava*, and 4% acetic acid treatment for *C. intestinalis* (3, 8, 9). The use of rock crab and green crab predation to provide biological control of tunicate populations on infested mussel socks has also been explored (7, 8). High-pressure washing is the mechanical mitigation method used most often by farmers in PEI to control *C. intestinalis* populations (10). This method can instantaneously knock off up to 100% of *C. intestinalis* (8); however, the effect is not long lasting, as the species quickly re-settles on the mussel socks, especially during the warm months when larval abundance and recruitment levels are at their peak (9, 11).

Investigating the efficacy (or effectiveness) of a variety of possible treatment scenarios is difficult in a field setting. Despite this some field trials have been carried out to compare the effectiveness of different mitigation strategies in terms of timing and frequency for colonial tunicates (10, 12) and *C. intestinalis* (13). However, these trials require considerable time to execute and are both cost- and labor- intensive. In light of this, computer-based simulation modeling, which allows for an evaluation of the likely impact of changes in treatment prior to implementation, is considered a useful alternative approach. The method has been proved effective in evaluating mitigation strategies to control sea lice population on salmon farms (14, 15).

In the context of computer-based simulation, effective algorithms to search for optimal solutions to complex problems (16) has been developed. Optimization software such us, OptQuest® Engine (17) provides a method to explore combinations of parameters (e.g., time, frequency, and efficacy of treatment) to quickly determine the best combination of variables that will result in achieving a desired objective (in the case under consideration, maximizing the reduction of *C. intestinalis* abundance).

We developed a mathematical model incorporating temperature-dependent growth and environmental carrying capacity to describe the population dynamics of *C. intestinalis* in areas with mussel production. The basic structure of this model was explained in an earlier paper (18), together with its sensitivity to various parameter and temperature changes; however, the application of this model to evaluate the effectiveness of mitigation strategies against *C. intestinalis* has not previously been explored. The objectives of this study were, therefore, (1) to evaluate the use of a mathematical model in exploring the optimal configuration of treatments given a set of pragmatic constraints in terms of a combination of timing and frequency to control *C. intestinalis* populations in areas with mussel production; (2) to evaluate the effectiveness of the preferred strategies suggested by the model under different temperature conditions; and (3) to assess the sensitivity of the modeled *C. intestinalis* population to variations in presumed levels of treatment efficacy under the preferred mitigation strategy.

## Materials and Methods

### *C. intestinalis* Population Dynamics Model

A previously described population dynamics model of *C. intestinalis* (18) was used to model the abundance of *C. intestinalis* in cases where treatment occurs, so that these could be compared to the situation in which no treatment (control) was administered. Briefly, this model consists of six compartments, representing five live life stages of *C. intestinalis*: egg (*E*), larva (*L*), recruit (*R*; the tadpole that settles on a surface and develops through a process of metamorphosis), juvenile (*J*; completely metamorphosed animal), spring adult (*A*_*sp*_; the animal that reaches its sexually mature size between May and September), and autumn adult (*A*_*au*_; the animal that reaches its sexually mature size between October and April). Two compartments are also set up to model dead stages [dead juvenile (*DJ*) and dead adult (*DA*)], so that all the surface-occupying stages (*N*_*SO*_) can be captured; which consist of these dead stages in addition to the *R, J, A*_*sp*_, and *A*_*au*_ stages.

The adult *C. intestinalis* spawns eggs when the water temperature is suitable (>4°C). These eggs are then fertilized and hatch into free-swimming larvae at water temperatures in the range from 8 to 26°C. The larvae find a substrate to settle on, undergo a process of metamorphosis, and become juveniles. The reproductive system develops as the juvenile grows, until it reaches sexual maturity, transforming the *C. intestinalis* into an adult which can produce sperm and eggs throughout its lifespan. The set of differential equations, describing the rates of change for each *C. intestinalis* life stage within the model, is shown in Table S1, while a description of the associated parameters is given in Table S2.

A dichotomous variable was used to control whether or not an adult could produce eggs based on the cut-off temperature of 4°C (Equation 1; Table S1). A similar approach was applied for spring and autumn adult compartments (Equations 5, 6; Table S1), where another dichotomous variable was created to define whether the modeled time was in the spring or autumn season. This allowed the model to assign animals from the juvenile stage to spring or autumn adult compartments, depending on the time of year in the model. The model was set to run for 220 days, with Day 1 being the 1st of May, and was initialized with an initial juvenile presence of 0.1 juvenile *C. intestinalis* per mussel sock (or approximately 1 juvenile per 10 mussel socks); all other life stages were initially set to zero.

### Parameters

The estimates of 19 parameters relating to the life cycle of *C. intestinalis* were adopted from the previous study (18). The details are presented in Table S2. In cases where a range of values had been reported, estimates were selected from uniform (for α) or triangular (for *L*_*L*_) distributions and the parameter values were updated for each modeled time step. The settlement rate of larvae is assumed to vary with the proportion of *N*_*SO*_*(t)* to environmental carrying capacity (*K*). *K* for a given bay is the maximum number of *C. intestinalis* that the system can accommodate on the total surface area (*a* in cm^2^) of all mussel socks in that bay. We adopted the estimates total surface area of 1.9 billion cm^2^ for Georgetown Harbor from Patanasatienkul et al. (18). A capacity adjusting factor γ*(a,t)*, representing the proportion of available surface area to the total surface area at time *t*, was used to adjust the settlement rate; as defined in Equation 9 (Table S1).

**Figure 1:**
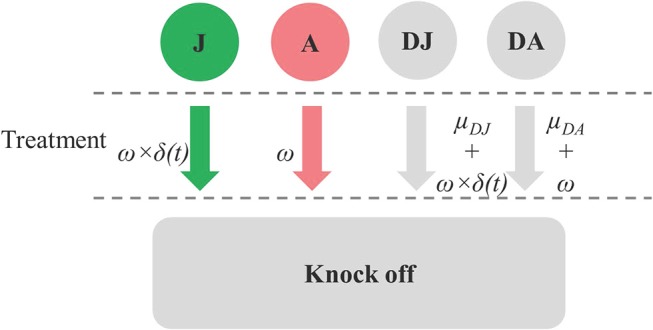
Diagram showing parameters related to drop-off rate from treatment effect (ω) on juvenile (J), adult (A), dead juvenile (DJ), and dead adult (DA) stages, the treatment effect adjusting factor for J and DJ [δ*(t)*], and natural drop-off of dead stages (μ_*DJ*_,and μ_*DA*_).

Space was released through a drop-off process whereby dead juveniles and adults were removed from the model at rates of μ_*DJ*_ and μ_*DA*_, respectively. Space was also released when treatments were applied. Each treatment was assumed to result in an instantaneously knock-down of ω for all the live and dead stages of juvenile and adult *C. intestinalis* from the treated mussel socks. Parameters related to treatment effect are shown in Figure 1. Treatment efficacy varies widely, depending on the method used; here, the default model assumed an instantaneous knock-down of 80%, based on the efficacy of the high-pressure washing method reported by Aren et al. (12). The treatment is less effective against the juvenile stages as the number of adults increases, as these protect the juvenile *C. intestinalis* from direct exposure to the treatment. Given that treatment efficacy for juveniles and dead juveniles depends on the proportion of live and dead adults to total abundance of juveniles and adults, this parameter was varied using an adjusting factor δ*(t)* (Equation 10 in Table S1).

### Treatment Strategies and Optimization Process

To find the optimal configuration of treatments given a set of pragmatic constraints, a range of treatment timings and frequencies were evaluated. Based on current practicalities affecting PEI mussel farmers, treatments could occurs as early as Day 61 (1st of July) up until Day 183 (31st of October) of the simulation. Frequency of treatment was allowed to vary from one to four times over a season, with a minimum duration of 14 days required between any pair of treatments; for example, if a treatment occurred at Day 61, the subsequent treatment could not occur until Day 75 at the earliest. The optimization process (with varied treatment intervals) was carried out separately for each treatment frequency during the course of the season. Treatment optimization, which had the goal of maximizing the reduction in *C. intestinalis* populations, was carried out within the AnyLogic® software package (19), using the built-in OptQuest® Engine (17). The search began at a lower bound (e.g., Day 61) with increments specified as a minimum step-size, and could range up to an upper bound (e.g., Day 183) in no specific search order (20). We used a step size of 7 days (i.e., assumed that treatments that occur within the same calendar week would be equally effective) for the optimization process to reduce the number of treatment combinations tested and the number of model runs, while still allowing for a relatively comprehensive exploration of possible treatment scenarios. The number of model runs varied based on the treatment frequency; for example, the optimization for the single treatment group required a lower number of model runs than was the case for multiple treatment interventions.

For each treatment scenario (i.e., combination of frequency and time of treatment), an objective value, used to measure the effectiveness of the intervention, was calculated as the sum of the differences in *N*_*SO*_ between the control (no treatment) and treatment scenarios across all modeled time points, using the identical parameter setting. Data were ranked in ascending order, based on this objective value (i.e., larger differences were given a higher rank), the percentiles of the objective value were computed. A treatment, in term of treatment timing, was considered to be among the “preferred mitigation strategies” when their objective value exceeded the 95th percentile for each of the four treatment groups, according to overall frequency of treatment (i.e., single, double, triple, and quadruple treatment groups).

### What-If Scenarios

Treatment optimization was explored under various scenarios to evaluate the impact of different treatment intervals, sea water temperatures, and levels of presumed treatment efficacy on the strategies that would yield the suitable results in controlling the populations of *C. intestinalis*.

#### Fixed Treatment Intervals

The advantage of treatment optimization with varied treatment intervals is that any combination of treatment times can be assessed; however, the preferred solutions suggested by the model may be unsuitable to put into practice, as farm activities are usually scheduled in a periodic manner. Therefore, fixed-treatment intervals were explored in the “what-if” scenarios.

The time of first treatment was varied between Day 61 (1st of July) and 183 (31st of October), and treatment frequency could range from one to four times, with fixed treatment intervals between each pair of treatments. Three treatment intervals (14, 28, and 56 days) were tested to assess their impact on the first treatment timing of treatment scenarios that were among the preferred mitigation strategies. Medians of treatment times within the preferred strategies were computed and used as a treatment combination for the evaluation of treatment effectiveness in the subsequent “what-if” analyses.

#### Temperature Conditions

Different sea water temperature conditions were used to create two what-if scenarios: long summer, and warm spring. The preferred mitigation strategy from the treatment optimization process with a fixed treatment interval, using the median of first treatment time with fixed treatment interval as a treatment setting, was evaluated by comparing the modeled *N*_*SO*_ under these temperature conditions. Furthermore, treatment optimization using varied treatment intervals was carried out to find the optimal configuration of treatments given a set of pragmatic constraints treatment strategy under each temperature scenario.

#### Treatment Efficacy

The influence of treatment efficacy assumptions on the modeled output (*N*_*SO*_) was assessed using a parameter variation method. The median of the first treatment timing and interval for the preferred mitigation strategies from the treatment optimization process with a fixed treatment interval was used as a treatment setting. The efficacy was then varied from 10 to 100% under the baseline temperature conditions, and the resulting objective values were compared to the default (80% treatment efficacy) scenario.

## Results

### Treatment Optimization With Varied Treatment Intervals

**Figure 2:**
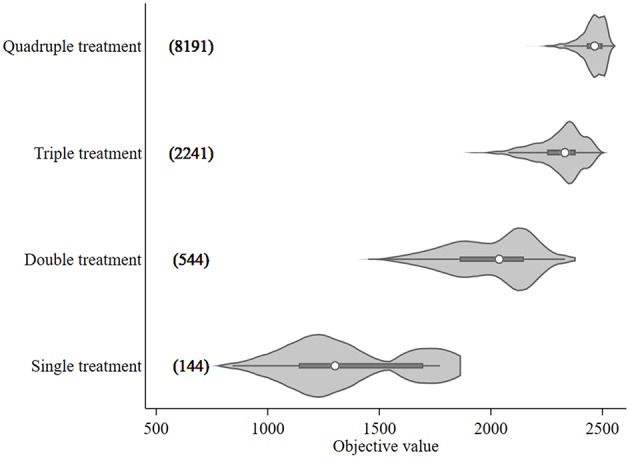
Violin plot of the objective values [sum of the differences in modeled surface occupying stages (*N*_*SO*_)] from *Ciona intestinalis* population dynamics model, and number of model runs (in bracket) for different treatment frequency groups. Circle, dark beam, and gray area define median, interquartile (IQR), and estimated kernel density, respectively. The whiskers extend to the lower and upper adjacent by 1.5 IQR.

The objective value, the sum of the differences between *N*_*SO*_ in the control and each treatment scenario, ranged from 842 to 2,552 for treatment optimization with a 14-day minimum treatment interval setting. These objective values, broken down by treatment frequency grouping, are shown in Figure 2 together with the numbers of model runs associated with each grouping. An increasing trend in objective value can be seen as more treatments are included. The median values for each treatment group were 1,302 (single treatment), 2,037 (double treatment), 2,332 (triple treatment), and 2,465 (quadruple treatment).

**Figure 3:**
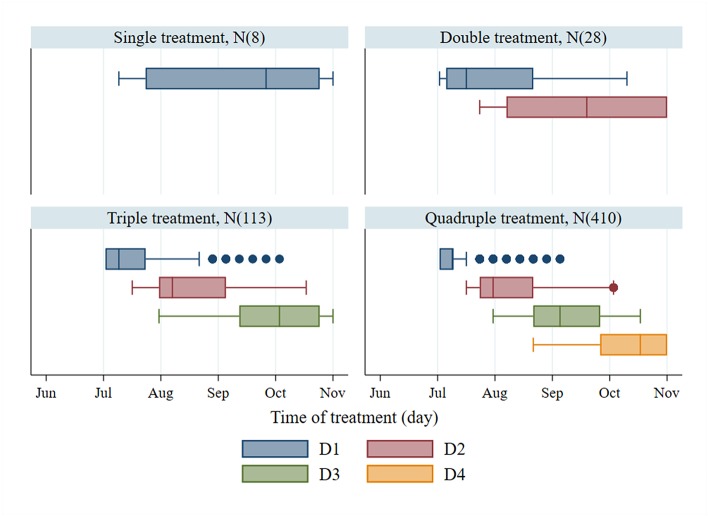
Treatment time of the first to fourth treatments (D1–D4) and number of observations (N) from the preferred mitigation strategies, which are the models with the objective values, ranking above the 95th percentile of the objective values for different treatment frequency groups.

The 95th percentile of the objective values for each treatment group were 1,772, 2,254, 2,451, and 2,524 for single, double, triple, and quadruple, respectively. Figure 3 illustrates the variation of treatment times for the preferred mitigation strategies by treatment frequency group. The treatment time for single treatments varied from mid-July to late October; however, the objective value associated with these single treatments were low (median value of 1,772) as compared to other treatments. The median of preferred treatment times for the double treatment group were in mid-July (Day 75) and mid-September (Day 138). The triple treatment group had median treatment times of early July (Day 68), early August (Day 96), and late-September (Day 152); while treatments given twice in July (Days 68 and 89), once in early September (Day 124), and once in mid-October (Day 166) were the medians for the quadruple treatment group.

### Treatment Optimization With Fixed Treatment Interval

**Figure 4:**
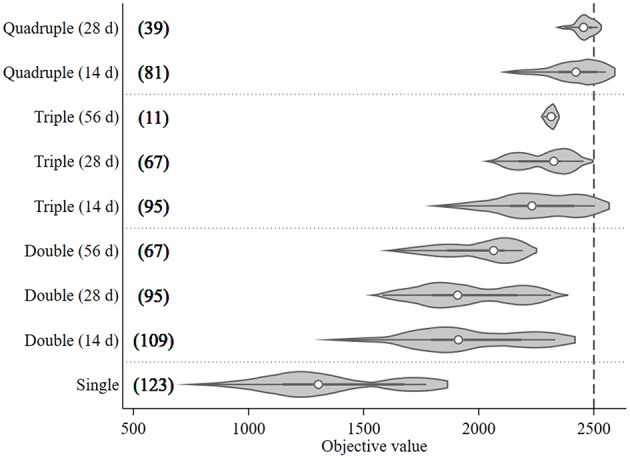
Violin plot of the objective values [sum of the differences in modeled surface occupying stages (*N*_*SO*_)] from *Ciona intestinalis* population dynamics model, and number of model runs (in bracket) for different treatment frequencies (one to four times) and fixed intervals (14, 28, and 56 days). Circle, dark beam, and gray area define median, interquartile (IQR), and estimated kernel density, respectively. The whiskers extend to the lower and upper adjacent by 1.5 IQR.

A total of 687 treatment scenarios were explored to find the optimal configuration of treatments given a set of pragmatic constraints when time of first treatment could be varied and the treatment intervals between each pair of treatments were fixed at 14, 28, or 56 days. The objective values and number of model runs associated with the assessed treatment frequency and interval are presented in Figure 4. The median objective value of the single treatment (1,300) was lower than the multiple treatment groups (around 2,000 for double treatment; 2,300 for triple treatment; and 2,400 for quadruple treatment groups). It was around half as high as the quadruple group. When comparing the outputs of different treatment intervals within the same treatment frequency group, the objective values of the preferred treatment (the right tail) for each treatment group tended to increase as the treatment intervals decreased (Figure 4). The triple treatment with a 14-day interval and quadruple treatments with 14- and 28-day intervals were the only three treatment groups that generated objective values exceeding the 95th percentile (2,500) when the objective values from all treatment groups were considered together. Among these three preferred treatment groups, quadruple treatment with a 14-day interval tended to have the highest median objective values and, therefore, its median treatment times were used as the treatment setting for the preferred mitigation strategy in the subsequent what-if scenarios section. The median treatment times for these three treatment combinations were Day 114 (triple treatment/14-day interval), 92 (quadruple treatment/14-day interval), and 81(quadruple treatment/28-day interval).

### Treatment Effectiveness Under Different Temperature Conditions

**Figure 5:**
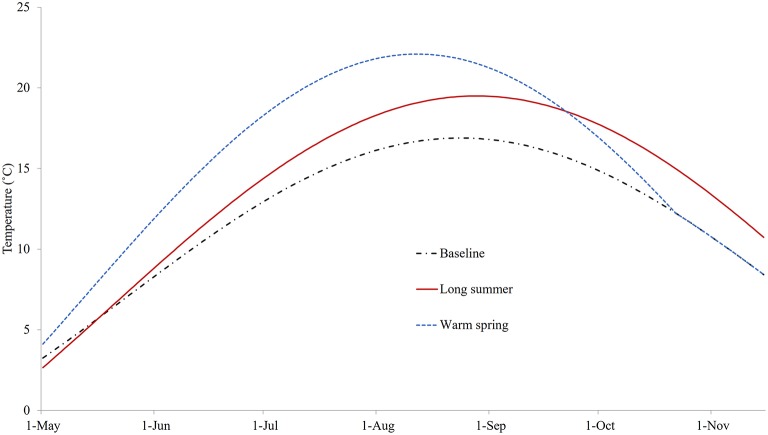
The temperatures for three different conditions: baseline (replicating temperature from Georgetown Harbor in 2008), long summer, and warm spring.

The three sea water temperature conditions explored are shown in Figure 5. The modeled temperature under the baseline condition was set to 3.3°C at the start of the model with a mean of 7.1°C. It peaked at a maximum of 16.9°C in late August, reached 8°C (the critical temperature for reproduction of *C. intestinalis*) at the end of May, and dropped below 8°C again in mid-November. In the case of the warm spring scenario, the maximum temperature started at 4.5°C and rose to around 5°C higher than the baseline for much of the summer before converging to the baseline profile by mid-October; while for the long summer scenario the maximum temperature was around 2°C higher from July, and remained so for around 2 months after the summer peak.

**Figure 6:**
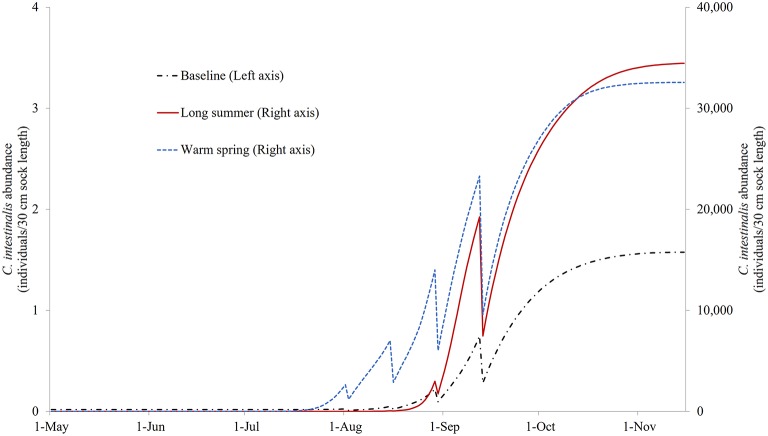
The modeled surface-occupying stages of *Ciona intestinalis* (*N*_*SO*_) with the preferred quadruple treatment (first treatment on Day 92 and repeated every 14 days) under baseline, long summer, and warm spring temperature conditions.

Figure S1 illustrates the modeled *N*_*SO*_ of the control model under the three different temperature profiles. It can be seen that based on these temperature changes, the growth of *C. intestinalis* is a few orders of magnitude greater in the absence of any treatment. Figure 6 illustrates the *N*_*SO*_ when the preferred (quadruple treatment/14 day interval, on Days 92, 106, 120, and 134 with treatment efficacy of 80%) mitigation strategy was carried out under the baseline, long summer, and warm spring temperature conditions. This preferred treatment strategy was obviously much less effective, leaving a large number of *N*_*SO*_ by the end of the year, when implemented under the long summer or warm spring conditions.

Median treatment times for the preferred mitigation strategies (i.e., treatments with objective values that exceeded 95th percentiles), explored under different temperature conditions, are presented in Tables 1, 2. The first treatment time of the multiple treatment groups (i.e., double, triple, and quadruple treatment) ranged from Day 68 to 75 for baseline temperature conditions, while the ranges were Day 75–121, and Day 82–124 for long summer, and warm spring conditions, respectively. The quadruple treatment group for the preferred treatment strategies tended to show higher objective values than other treatment frequency groups, explored under the same temperature conditions.

**Table 1:** Comparison of the preferred mitigation strategies (i.e., treatment combinations with objective values ranking above the 95th percentile for each treatment frequency group) under baseline temperature condition.

**Temperature condition**	**Treatment frequency**	***N***	**Objective value**	**D1**	**D2**	**D3**	**D4**
Baseline	1	8	1,772 (0)	145	–	–	–
	2	28	2,309 (24)	75	138	–	–
	3	113	2,464 (12)	68	96	152	–
	4	410	2,533 (7)	68	89	124	166

*Details include number of model runs (N), objective values [mean (standard deviation)], and median of treatment time (D1–D4). Note that an objective value is the sum of the difference in surface-occupying stage between control and treatment, and Day 1 was set to the 1st of May*.

**Table 2:** Comparison of the preferred mitigation strategies (i.e., treatment combinations with objective values ranking above the 95th percentile for each treatment frequency group) under long summer and warm spring temperature conditions.

**Temperature condition**	**Treatment frequency**	***N***	**Objective value**	**D1**	**D2**	**D3**	**D4**
Long summer	1	8	947,132 (0)	163	–	–	–
	2	28	1,317,279 (29,064)	121	173	–	–
	3	113	1,565,439 (71,809)	96	145	173	–
	4	410	1,776,395 (51,952)	75	117	145	180
Warm spring	1	8	1,174,553 (0)	142	–	–	–
	2	28	1,592,415 (33,666)	124	173	–	–
	3	113	1,896,000 (51,479)	96	138	173	–
	4	410	2,167,043 (38,210)	82	117	145	173

*Details include number of model runs (N), objective values [mean (standard deviation)], and median of treatment time (D1–D4). Note that an objective value is the sum of the difference in surface-occupying stage between control and treatment, and Day 1 was set to the 1st of May*.

### Sensitivity to Treatment Efficacy Variation

**Figure 7:**
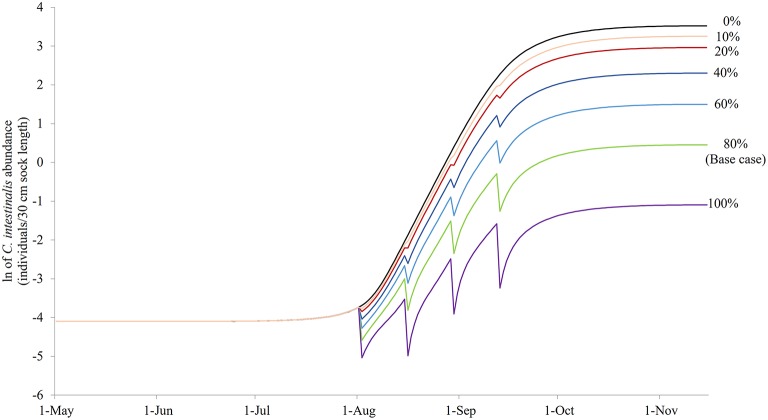
The modeled surface-occupying stages of *Ciona intestinalis* [*N*_*SO*_ (individuals per 30 cm sock length)] in natural-logarithm unit with the preferred quadruple treatment (first treatment on Day 92 and repeated every 14 days) under different treatment efficacy assumptions with 80% treatment efficacy as the base case.

**Figure 8:**
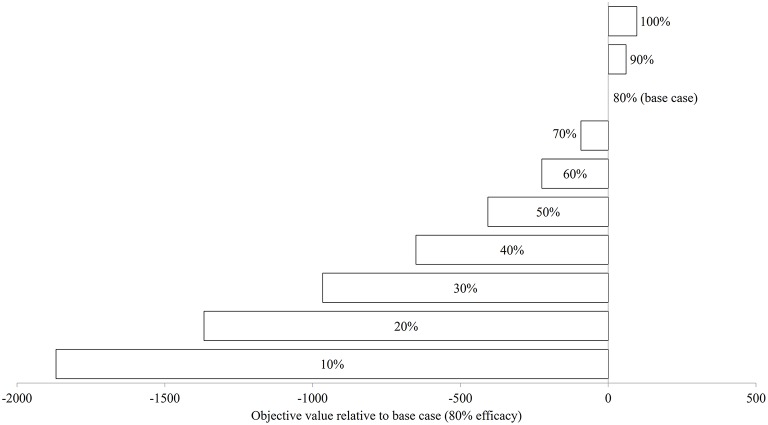
The changes in objective value (sum of the differences in surface-occupying stage between control and treatment) relative to base case (80% treatment efficacy) of *Ciona intestinalis* population dynamics under baseline temperature condition.

Figure 7 illustrates the modeled *N*_*SO*_ in natural-logarithm unit when treatments were carried out at Day 92, 106, 120, and 134 with treatment efficacy varying from 0% (control or no treatment) to 100% under the baseline temperature condition, while Figure 8 presents the changes in the objective values of different treatment efficacies relative to the base case scenario. The actual objective values can be seen in Figure S2. The modeled *N*_*SO*_ for the control gradually increased from late July until late August with a rapid increase in early September, reaching a plateau at an abundance of over 30 individuals per 30 cm sock (Figure 7). When treatments were implemented, the modeled *N*_*SO*_ broadly followed the output from the control scenario, with lower levels for the abundance of *C. intestinalis* and objective value. As the treatment efficacy increased, the modeled *N*_*SO*_ decreased, with the abundance that varied from 0.3 (for 100% efficacy) to 26 individuals per 30 cm sock (for 10% efficacy). In contrast, the objective value increased considerably from 608 (for 10% efficacy) to 2,573 (for 100% efficacy), as the treatment efficacy increased (Figure S2). The variation of treatment efficacy between 10 and 60% caused a moderate change in objective value as compared to the outcome from the base case scenario, but the objective values showed only minimal variation over the rest of the treatment efficacy range of 70–100% (Figure 8 and Figure S2).

## Discussion

### Treatment Optimization

The treatment optimization from the model suggests that the multiple treatment should start early in July, assuming baseline temperature conditions. This result agrees with a field trial study in PEI in 2008 (13, 21), which found that the best strategy to reduce tunicate fouling, regardless of mussel productivity, was to use three or four treatments starting in July. The model also suggested including one late treatment (>Day 135 or mid-September) in all strategies to achieve effective control of *C. intestinalis* populations, which is reasonable as this late treatment will clean up the mussel socks around the end of the season when *C. intestinalis* enters its slow reproduction period (6), and provide limited time for the recruiting stage to re-settle on the socks.

The treatment optimization also suggests that the mitigation strategies with higher treatment frequency appear to be more effective than the less frequent strategies, especially, under the warm spring condition, which shows the highest objective value for the quadruple treatment group. This is in agreement with the results from a study (10) that evaluated the effectiveness of different treatment frequencies to control colonial tunicates. The result is also consistent with the result from a treatment trial (7), using vinegar and lime, which reported that double treatments resulted in larger reductions of *C. intestinalis* biomass than single treatments. Furthermore, when considering results from the optimization with fixed treatment intervals, the objective values among the preferred strategies (the right tails) of each treatment group showed an increasing trend as the time intervals between treatments decreased. This suggests that after taking into account the timing of first treatment, the mitigation strategy using multiple treatments may be more effective when the additional treatments are implemented shortly after the previous treatment; however, this also means adding the cost of the extra treatment. More information is needed to justify the cost-benefit of this approach.

### Treatment Effectiveness Under Different Temperature Conditions

When treatment timings based on the preferred mitigation strategies from the baseline temperature scenario were applied to the long summer and warm spring scenarios, their ability to control the population was poor, since the effectiveness of a treatment to control *C. intestinalis* populations depends greatly on when the treatment is implemented. If it is carried out during the warm months, when the temperature is suitable for the reproduction of *C. intestinalis* (6, 11), the treatment may not be so effective. This is because most treatments remove biofouling species from mussel sock surfaces (3), which indirectly facilitates the regrowth of the tunicate populations by increasing the surface availability for the larval stage to settle on the mussel socks. Therefore, a combination of treatment time and frequency that is considered the preferred mitigation strategy under one temperature condition may not perform well when implemented under different temperature conditions.

Given that the preferred mitigation strategies based on baseline conditions did not work well for other modeled temperature profiles, treatment optimizations were also attempted for the warmer temperature conditions (i.e., long summer and warm spring). The preferred time of the first treatment under these warmer conditions appeared to be later in the season than was suggested for the baseline scenario. As discussed above, temperature plays an important role in the development of *C. intestinalis* populations (6) and should be taken into careful consideration when exploring the mitigation strategies to control this biofouling species.

### Sensitivity to Treatment Efficacy Variation

As might be expected, an increase in treatment efficacy resulted in an increase in the objective value, which is after all an indication of successful treatment. The increased effectiveness of treatments rose substantially up to an efficacy level of ~70%. Thereafter, any increase in effectiveness of the treatment did not result in a significant improvement in terms of reduced abundance of *C. intestinalis*. This suggests that a minimum treatment efficacy of 70% should be sufficient to sustain control of *C. intestinalis* populations. However, this result should be interpreted with caution, as the objective value in this study is based on the abundance of aggregated surface-occupying stages, and has not accounted for the difference in the size/ weight of life stages (e.g., juvenile and adult), which is associated with sock attachment strength of mussels. Although biomass is an appropriate value to measure the effectiveness of a treatment, it was not applied to this study due to limited data available with which to parameterize the model. Further research should explore the effectiveness of treatments, using biomass, before more conclusive statements regarding treatment efficacy are made.

In conclusion, this mathematical model provides a means to explore the optimal configuration of treatments given a set of pragmatic constraints, and can also be used to assess approaches to reduce *C. intestinalis* population levels under different temperature conditions. The model provides flexibility to explore the effectiveness of different treatment scenarios, e.g., varying the time of treatment, treatment frequency, and treatment efficacy. This model can therefore be used as a tool to develop better mitigation strategies to control populations of aquatic invasive species under different environmental conditions and to help improve bay management plans for the mussel industry. Future models should include information on *C. intestinalis* biomass, and cost effectiveness, to find the best mitigation strategies for controlling *C. intestinalis* populations without compromising mussel yield.

## Author Contributions

TP, JS, JD, and CR contributed conception and design of the study. TP performed the model building, statistical analysis, and wrote the first draft of the manuscript. All authors contributed to manuscript revision, read, and approved the submitted version.

### Conflict of Interest Statement

The authors declare that the research was conducted in the absence of any commercial or financial relationships that could be construed as a potential conflict of interest.
