# Supplementary material for: The Application of a Mathematical Model to Evaluate the Effectiveness of Control Strategies Against Ciona intestinalis in Mussel Production
Source: Front Vet Sci. 2019 Aug 20;6:271. doi: 10.3389/fvets.2019.00271 (PMC6710327; doi:10.3389/fvets.2019.00271)
Supplement: Supplementary file 1 [file Data_Sheet_1.pdf]

Front. Vet. Sci. 6:271. doi:10.3389/fvets.2019.00271

Supplementary Material

The Application of a Mathematical Model to Evaluate the Effectiveness of Control Strategies against *Ciona intestinalis* in Mussel Production

Thitiwan Patanasatienkul*, Javier Sanchez, Jeff Davidson, and Crawford W. Revie

*** Correspondence:** Dr. Thitiwan Patanasatienkul: thitiwan.patanasatienkul@gmail.com

Table S1. Mathematical equations for the *Ciona intestinalis* population dynamics model used to evaluate effectiveness of various mitigation strategies.

Mathematical equations	Number
$\frac{dE(t)}{dt} = \frac{(x \times a) \times F_f(T^\circ)}{G_{SI}} \times \left(A_{sp}(t) + A_{au}(t)\right) - \frac{F_h(T^\circ)}{G_E(T^\circ)} \times E(t) - \frac{E(t)}{L_E}; x = \begin{cases} 0, T^\circ < 4°\mathrm{C} \\ 1, T^\circ \geq 4°\mathrm{C} \end{cases}$	*Eq. 1*
$\frac{dL(t)}{dt} = \frac{F_h(T^\circ)}{G_E(T^\circ)} \times E(t) - \frac{F_s(T^\circ)}{G_L(T^\circ)} \times \gamma(a,t) \times L(t) - \frac{L(t)}{L_L}$	*Eq. 2*
$\frac{dR(t)}{dt} = \frac{F_s(T^\circ)}{G_L(T^\circ)} \times \gamma(a,t) \times L(t) - \frac{F_m(T^\circ)}{G_R} \times R(t) - \frac{1-F_m(T^\circ)}{G_R} \times R(t)$	*Eq. 3*
$\frac{dJ(t)}{dt} = \frac{F_m(T^\circ)}{G_R} \times R(t) - \frac{J(t)}{G_J(T^\circ)} - m_J \times J(t) - \omega \times \delta(t) \times J(t)$	*Eq. 4*
$\frac{dA_{sp}(t)}{dt} = \frac{J(t)}{G_J(T^\circ)} \times y - \frac{A_{sp}(t)}{L_{A_{sp}}} - \omega \times A_{sp}(t); y = \begin{cases} 0, t > 120 \\ 1, t \leq 120 \end{cases}$	*Eq. 5*
$\frac{dA_{au}(t)}{dt} = \frac{J(t)}{G_J(T^\circ)} \times (1-y) - \frac{A_{au}(t)}{L_{A_{au}}} - \omega \times A_{au}(t); y = \begin{cases} 0, t > 120 \\ 1, t \leq 120 \end{cases}$	*Eq. 6*
$\frac{dDJ(t)}{dt} = m_J \times J(t) - (\mu_{DJ} + \omega \times \delta(t)) \times DJ(t)$	*Eq. 7*
$\frac{dDA(t)}{dt} = \frac{A_{sp}(t)}{L_{A_{sp}}} + \frac{A_{au}(t)}{L_{A_{au}}} - (\mu_{DA} + \omega) \times DA(t)$	*Eq. 8*
$\gamma(a,t) = 1 - \frac{N_{SO}(t)}{K \times a}$	*Eq. 9*
$\delta(t) = 1 - \frac{A_{sp}(t)+A_{au}(t)+DA(t)}{A_{sp}(t)+A_{au}(t)+DA(t)+J(t)+DJ(t)}$	*Eq. 10*

Table S2. Parameter definitions and estimates for *Ciona intestinalis* population dynamics model that includes a treatment effect. (Temperature-dependent parameters are marked with an asterisk.)

Parameter	Description	Value	Unit
$G_E(T^{\circ})$	Development time of egg*	0.51 – 2.63	day
$G_L(T^{\circ})$	Development time of larva*	0.31 - 10	day
G_R	Development time of recruit	12	day
$G_J(T^{\circ})$	Development time of juvenile*	30 - 90	day
G_{SI}	Spawning interval	3	day
α	Number of eggs laid per spawning	1,000 – 1,500 (uniform)	egg
$F_f(T^{\circ})$	%Fertilization*	0 – 85	%
$F_h(T^{\circ})$	%Hatchability*	0 - 85	%
$F_s(T^{\circ})$	%Settlement*	0 - 65	%
$F_m(T^{\circ})$	%Metamorphosis*	0 – 80	%
L_E	Lifespan of egg	1.25	day
L_L	Lifespan of larva	0.25-1.5 (triangular: min, (min+max)/2, max)	day
L_{Asp}	Lifespan of spring-adult	150	day
L_{Aau}	Lifespan of autumn-adult	180	day
m_J	% Mortality of juvenile	0.11	%
μ_{DJ}	% daily drop-off of dead juvenile	0.05	%
μ_{DA}	% daily drop-off of dead adult	0.05	%
ω	Treatment efficacy**	80 (default) (0-100 sensitivity test)	%
K	Environmental carrying capacity	40	individual·cm^{-2}

* designates temperature dependent parameter.

** The effect depends on the proportion of live and dead adult on the surface.

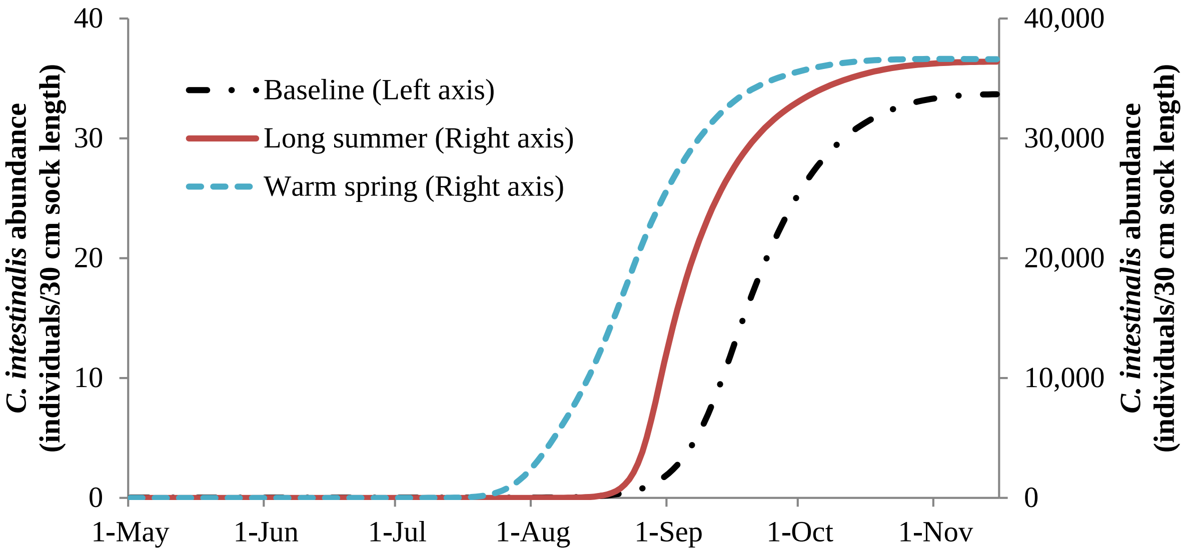

Figure S1. The modelled surface-occupying stages of *Ciona intestinalis* (N_{SO}) when no treatment was administered (control) under baseline, long summer, and warm spring temperature conditions.

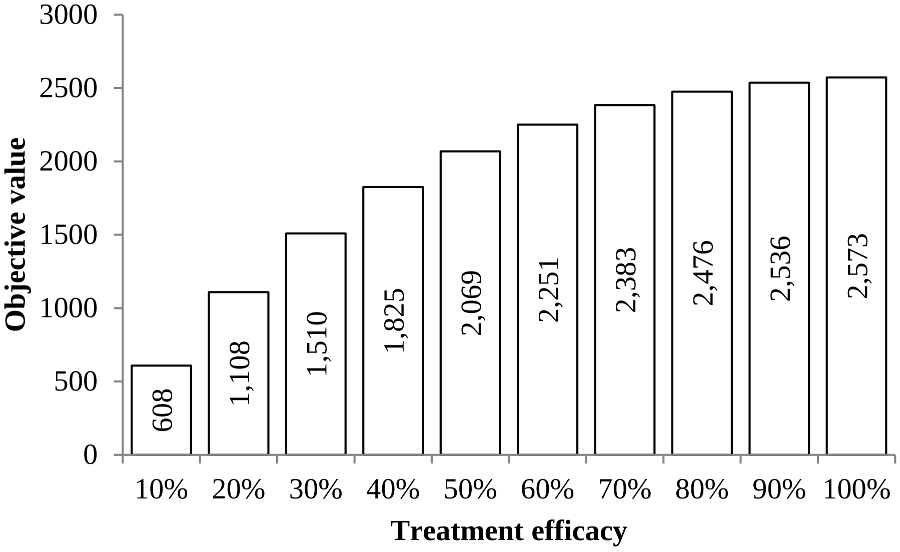

Figure S2. The objective values (sum of the difference in surface-occupying stage of *Ciona intestinalis* between control and treatment models) for the variation of treatment efficacy from 10% to 100%.
